# Effect of Surface Plasmon Coupling to Optical Cavity Modes on the Field Enhancement and Spectral Response of Dimer-Based sensors

**DOI:** 10.1038/s41598-017-11140-0

**Published:** 2017-09-05

**Authors:** Salma Alrasheed, Enzo Di Fabrizio

**Affiliations:** King Abdullah University of Science and Technology, PSE and BESE Divisions, Thuwal, 23955-6900 Saudi Arabia

## Abstract

We present a theoretical approach to narrow the plasmon linewidth and enhance the near-field intensity at a plasmonic dimer gap (hot spot) through coupling the electric localized surface plasmon (LSP) resonance of a silver hemispherical dimer with the resonant modes of a Fabry-Perot (FP) cavity. The strong coupling is demonstrated by the large anticrossing in the reflection spectra and a Rabi splitting of 76 meV. Up to 2-fold enhancement increase can be achieved compared to that without using the cavity. Such high field enhancement has potential applications in optics, including sensors and high resolution imaging devices. In addition, the resonance splitting allows for greater flexibility in using the same array at different wavelengths. We then further propose a practical design to realize such a device and include dimers of different shapes and materials.

## Introduction

Plasmonics is an important branch of nanophotonics and is the study of the interaction of electromagnetic fields with the free electrons in a metal at metallic/dielectric interfaces or in small metallic nanostructures. The electric component of an exciting electromagnetic field can induce collective electron oscillations known as surface plasmons. Such oscillations lead to the localization of the fields that can be at sub-wavelength scale and to its significant enhancement relative to the excitation fields. These two characteristics of localization and enhancement are the main components that allow for the guiding and manipulation of light beyond the diffraction limit. Examples of surface-plasmon based devices include biosensors, high-resolution imaging, solar cells, waveguides, data storage, light emitting devices etc.^1–9^.

One of the main powerful methods used in sensors is surface enhanced Raman spectroscopy (SERS), which is the enhancement of the Raman signal achieved by placing Raman-active molecules in the vicinity of the near field of a metallic nanostructure^10–12^. Single and few molecules detection is a desired feature and can be achieved through a higher enhancement and localization of the electric field^10–14^. Closely spaced plasmonic nanoparticles such as hemisphere dimers and dimers of other shapes form nano-sized gaps with strong field enhancement and localization within a few cubic nanometers, and are knows as hot spots^15–19^. The Raman signal is proportional to the fourth power of the electromagnetic field enhancement factor $({|{E}_{loc}|}^{4}/{|{E}_{o}|}^{4})$, therefore a higher local field enhancement $|{E}_{loc}|$ in the hot spot greatly enhances the Raman signal, and its localization in a small gap enables the detection of few molecules deposited there. Many different configurations were used to realize such features of high enhancement and localization including self-similar chains (SSCs) nanolense^14^, nanocuboids^5^, nanospheres^20, 21^, nanorods^22^, and nanoparticles clusters^23^.

One of the major problems in plasmonics is the large linewidth of plasmon resonances due to the large radiative damping of the metal^16, 24, 25^. Several methods have been used to decrease the linewidth and enhance the quality factor of the resonance. This requires coupling the broad LSP resonance to a system with a narrow resonance. Examples of such methods include far-field diffractive coupling^24–31^, coupling with a quadruple resonance^32^, coupling with waveguide modes^33^ and coupling with a photonic microcavity^34–49^. In this paper, we propose an approach to narrow the plasmon linewidth and produce a large near-field enhancement in the gap (hot spot) of a silver plasmonic hemispherical dimer. The coupling mechanism involves the dimers with the collective resonance mode of the array, enhanced by far-field diffractive coupling, and the resonant modes of a Fabry-Perot (FP) cavity. First we demonstrate, using a hemispherical silver dimers array embedded in a FP cavity, an anticrossing in the reflection spectra and a Rabi splitting of 76 meV due to the strong coupling between the silver dimers array collective resonance mode and the optical cavity’s second mode. The resulting near-field intensity is 24% enhanced following the introduction of the FP cavity (with a maximum enhancement factor of $|{E}_{loc}|/|{E}_{0}|=252.6$) compared to the bare dimers array without the cavity. In addition, the resonance splitting allows for greater flexibility in using the same array at different wavelengths. We then further propose a practical design to realize such a device to maximize the near-field enhancement and include dimers of different shapes and materials, where we show that up to 2-fold increase in enhancement can be achieved for a certain configuration. The computational method used is the finite-difference time-domain method (FDTD).

## Structure and Design

**Figure 1:**
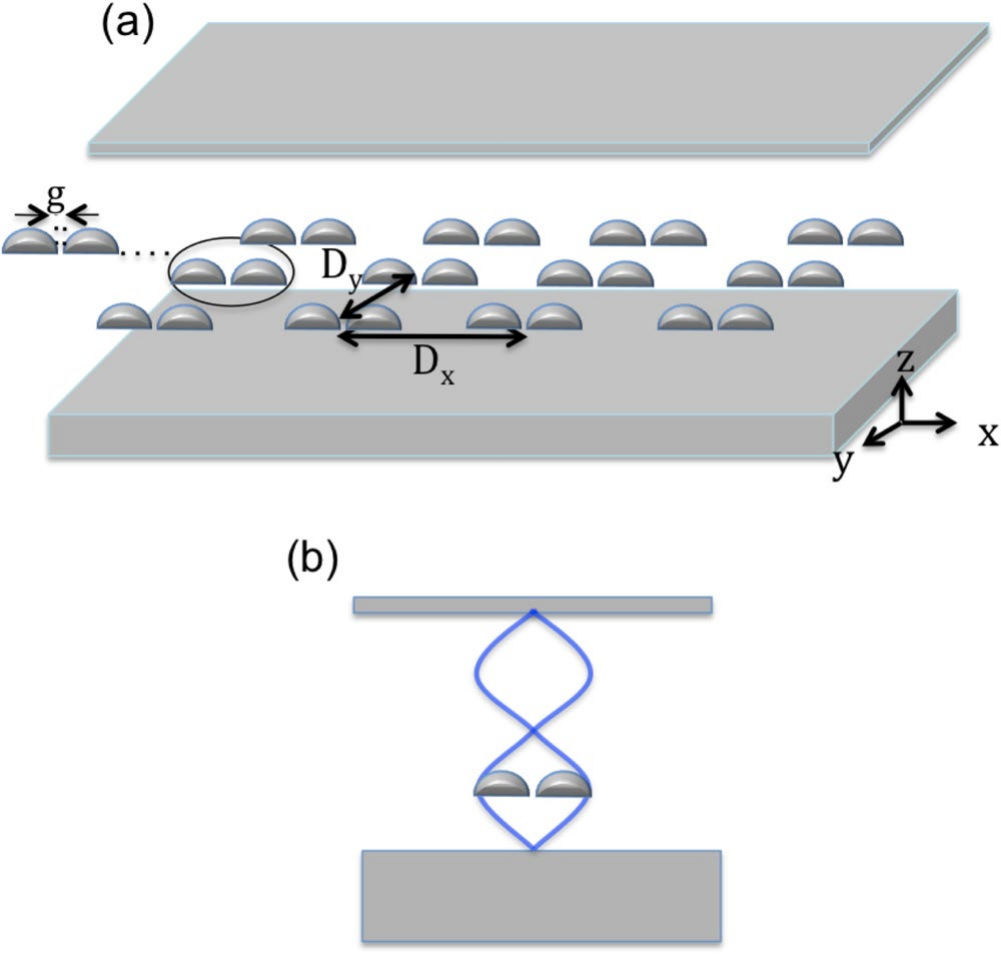
(**a**) Schematic representation of a hemispherical dimers array placed within an FP cavity. Each hemisphere has a radius of 45 nm and the gap between the hemispheres is 5 nm. Both the dimer and the FP mirrors are silver where the complex refractive index is taken from the data of Palik (0–2 μm)^50^. The surrounding medium is air (index of refraction = 1). The dimers array is in the x-y plane with a periodicity (dimer center-to-center) of 400 nm and 420 nm in the *x* and *y* directions respectively. The FP upper mirror is of a thickness of 15 nm as to allow for the light from the source to pass through it. The lower mirror’s thickness is 500 nm. The total cavity’s length (**d**) is 478 nm (separation between the two mirrors). A TM plane wave source with a wavelength ranging from 450 nm to 720 nm, placed above the FP cavity (above the 15 nm thick mirror), illuminates the array at normal incidence along the *z* direction. (**b**) Since we are using the second mode of the FP cavity, and we need the position where the electric field of the FP cavity standing wave to be a maximum, we place the dimers array at quarter the cavity length above the lower mirror.

**Figure 2:**
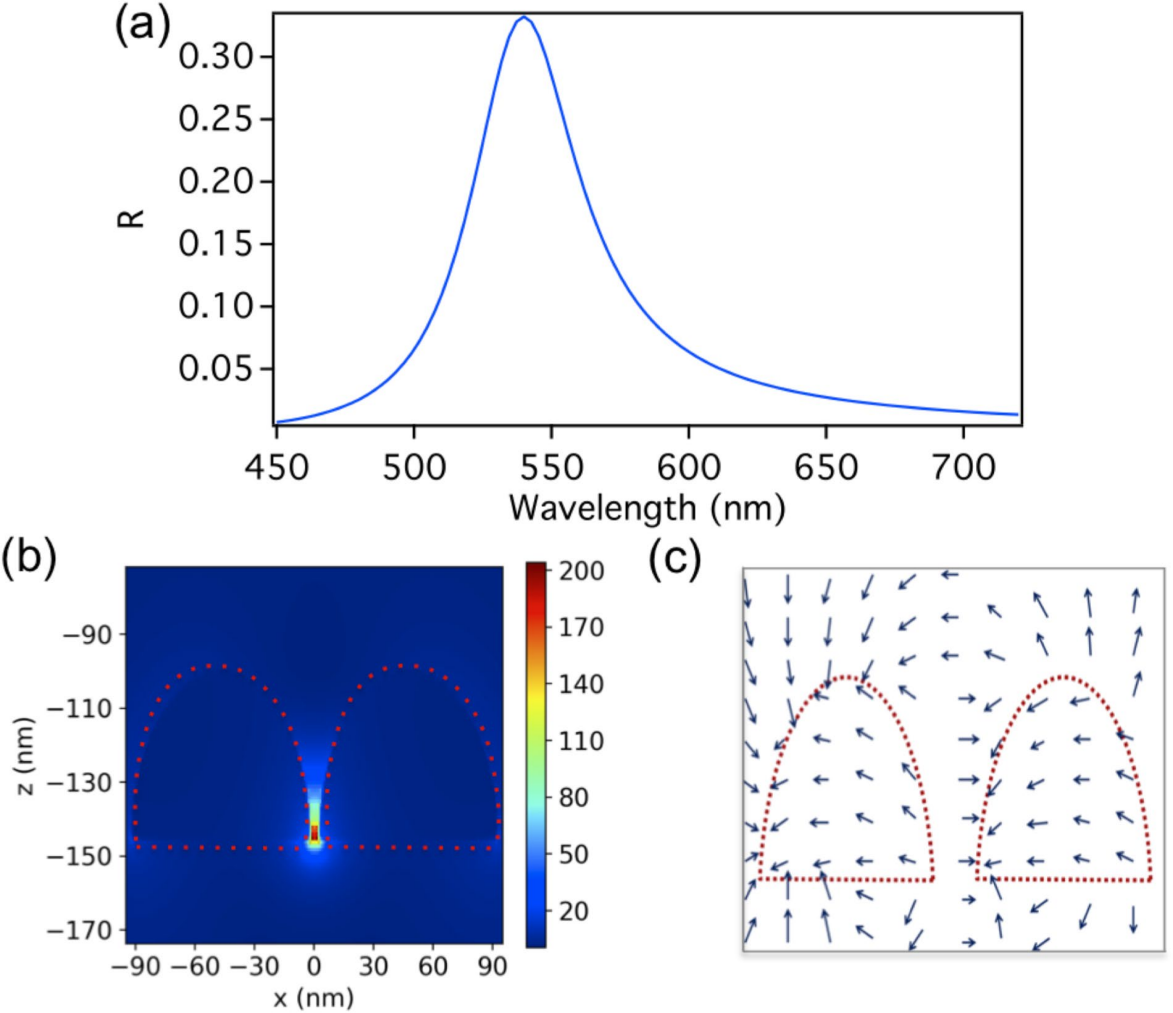
(**a**) The reflection spectrum of the bare dimers array without the FP cavity. A reflection maximum is observed at λ = 540 nm, which is due to the excitation of the dimer’s longitudinal LSP mode enhanced by the far-field diffraction coupling. (**b**) Near electric field intensity distribution at the reflection peak of λ = 540 nm for the bare dimers array for a unit cell (containing one dimer) where an enhancement factor of $|{E}_{loc}|/|{E}_{0}|=204$ is observed. (**c**) The electric field vectors represented by the arrows show the longitudinal LSP mode of the dimer.

In our first model, a hemispherical silver dimers periodic array is placed within a FP cavity as shown in Fig. 1(a). Each hemisphere has a radius of 45 nm and the gap between the hemispheres is 5 nm. Both the dimer and the FP mirrors are silver where the complex refractive index is taken from the data of Palik (0–2 μm)^50^. The surrounding medium is air (index of refraction = 1). The dimers array is in the x-y plane with a periodicity (dimer distance is referred to center-to-center) of 400 nm and 420 nm in the *x* and *y* directions respectively. Note that the dimer size and the periodicity of the array are chosen such that the collective resonance wavelength of the array matches that of the FP cavity mode (in this case the second order cavity mode). The FP upper mirror is of a thickness of 15 nm as to allow for the light from the source to pass through it (the transmissions ranges from 71% to 43% as the wavelength changes from 450 nm to 720 nm). The lower mirror’s thickness is 500 nm (zero transmission). The total cavity’s length (d) is 478 nm (separation between the two mirrors). A TM plane wave source with a wavelength ranging from 450 nm to 720 nm, placed above the FP cavity (above the 15 nm thick mirror), illuminates the array at normal incidence along the *z* direction. Since we are using the second mode of the FP cavity, and we need the position where the electric field of the FP cavity standing wave has a maximum, we place the dimers array at the cavity quarter length above the lower mirror as shown in Fig. 1(b). Note that the first order mode is at 1115 nm and is far from the dimer resonance. Perfectly matched layer boundary conditions (PML) where used above and below the array in the z-direction to absorb scattered waves at the boundaries of the simulation domain. The mesh accuracy is set to 3, corresponding to a λ/14 step size. A mesh override region of 0.1 nm in all three directions is used around the dimer for a better resolution of the gap’s near-field enhancement. To simulate an infinite array of the unit cell, periodic boundary conditions are used in both *x* and *y* directions. The periodicity in *x* and *y* allows for far-field diffraction coupling^24–31^ between the dimers, where the net acting field on every dipole includes the incident field and the sum of the radiation fields from all other dipoles, this substantially modifies the particle polarizability relative to the single particle resonance, resulting in enhanced scattering intensity of the array, in addition to narrowing the plasmon linewidth. This is accompanied by enhancing the near field intensity of the individual dimer^24–31^. We use such enhanced peak due to diffractive coupling, to further couple it with the FP cavity. For our finite-difference time-domain method (FDTD), we use the commercial software (Lumerical). The reflection spectrum of the bare dimers array without the FP cavity is shown in Fig. 2(a). A reflection maximum is observed at λ = 540 nm, which is due to the excitation of the dimer’s longitudinal electric LSP mode (As can be seen from the electric field vector arrows in Fig. 2(c)) enhanced by the far-field diffraction coupling. Figure 2(b) shows the corresponding near electric field intensity distribution at the reflection peak for the bare dimers array for a unit cell (containing one dimer) where an enhancement factor of $|{E}_{loc}|/|{E}_{0}|=\,204$ is observed.

**Figure 3:**
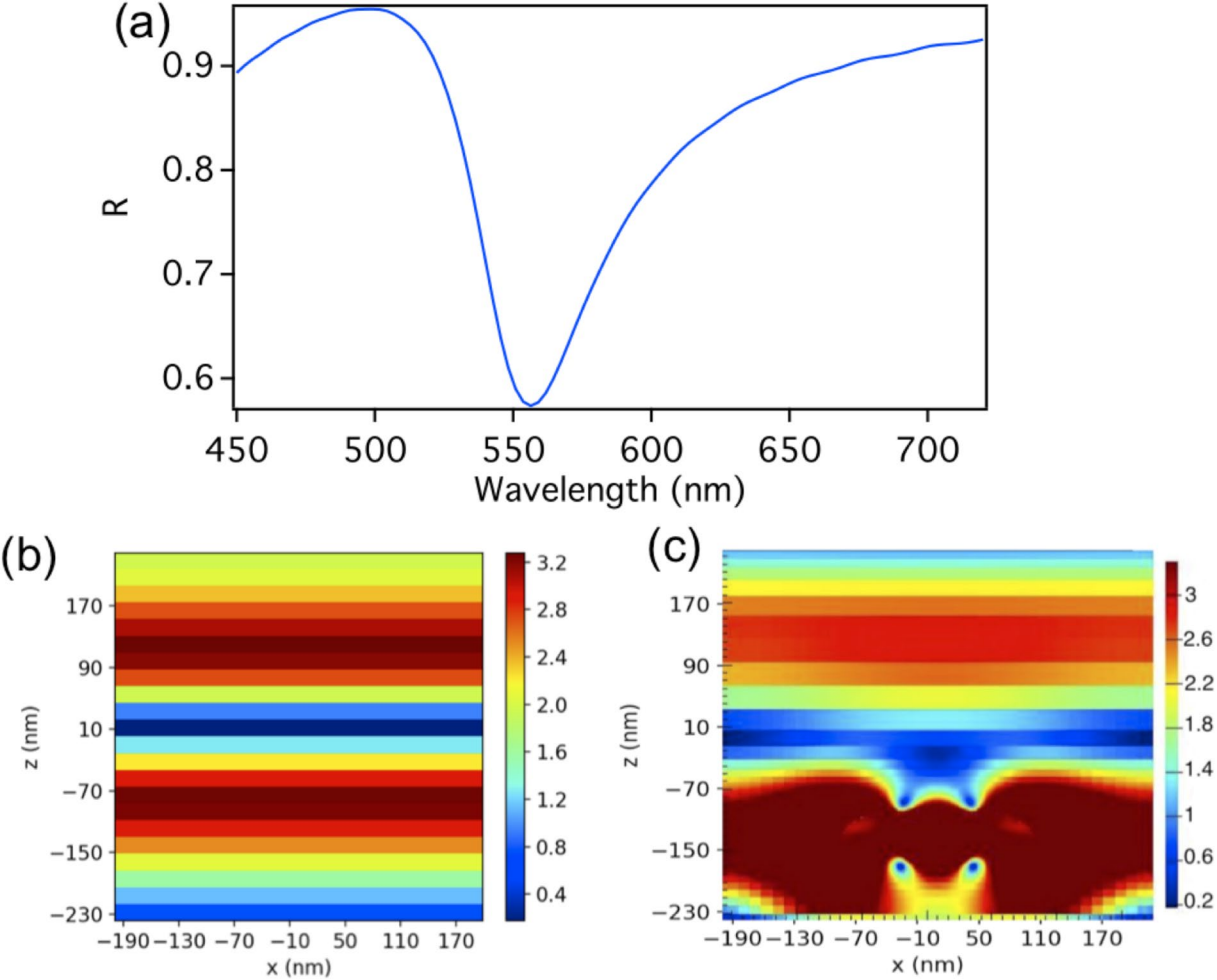
(**a**) The reflection spectra of the FP cavity of a total length of d = 478 nm without embedding the dimers array. A reflection dip is observed at λ = 556.4 nm resulting from the excitation of the second order cavity mode. (**b**) The electric field intensity distribution of the FP standing wave of the second order mode. (**c**) The electric field intensity of the FP second order standing wave as it interacts with the dimer (for a unit cell) when the dimer array is placed within the cavity. Note that the electric field intensity is normalized here to 3.3 as to make the interaction more clear, however the actual field intensity is 228.3 V/m and 252.6 V/m at the dimer gap for the higher and lower energy modes respectively as shown in Fig. 4.

**Figure 4:**
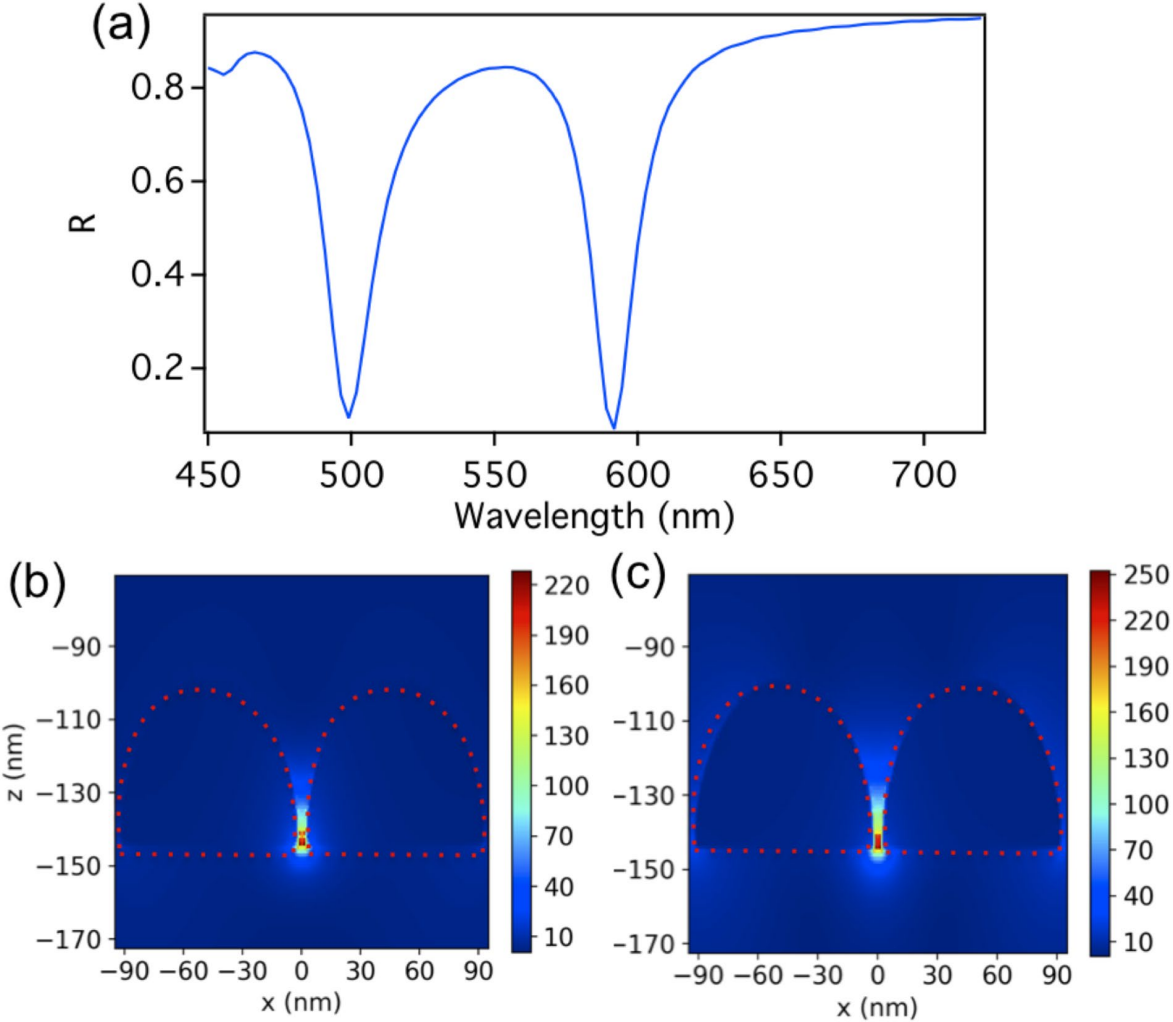
(**a**) Shows the dimers array mode splits into two hybridized modes of narrow bandwidth when the array is placed within the cavity of a total length of d = 478 nm. (**b**) and (**c**) shows the corresponding near electric field intensity enhancement of the unit cell of the dimers array (containing one dimer), when embedded in a FP cavity, for the higher and lower energy modes respectively (at a wavelength of 499 nm and 591.8 nm respectively). An enhancement factor of $|{E}_{loc}|/|{E}_{0}|=228.3$ is shown at the gap of the dimer for the higher energy hybridized mode in (**b**), while for the lower energy hybridized mode in (**c**); the near- field enhancement is $|{E}_{loc}|/|{E}_{0}|=252.6$ at the gap of the dimer. The full width half-maximum (FWHM) for each of the higher and lower energy dips is 19 nm and 15 nm respectively.

A further step in our study is the insertion of the array in a FP cavity. In Fig. 3(a) we show the reflection spectra of the FP cavity of a total length of d = 478 nm without embedding the dimers array. A reflection dip is observed at λ = 556.4 nm which results from the excitation of the second order cavity mode. Figure 3(b) shows the electric field intensity distribution for the FP standing wave of the second order mode. The dimer array is then placed within this cavity. Since we are using the second mode of the FP cavity, and we need the position where the electric field of the FP cavity standing wave to be a maximum, we place the dimer array at quarter the cavity length above the lower mirror as shown in Fig. 1(b). Figure 3(c) shows the electric field intensity distribution of the FP second order standing wave as it interacts with the dimer (for a unit cell) when the dimer array is placed within the cavity. Note that the field intensity is normalized to an electric field intensity of 3.3 V/m as to make the interaction between the dimer and the cavity more clear, however the actual field intensity is 228.3 V/m and 252.6 V/m at the dimer gap for the higher and lower energy modes respectively as shown in Fig. 4.

Further, in Fig. 4(a) it is shown the dimers array mode that splits into two hybridized modes of narrow bandwidth when the array is placed within the cavity. Figure 4(b) and (c) shows the corresponding near electric field intensity enhancement of the unit cell of the dimers array, when embedded in a FP cavity, for the higher and lower energy modes respectively (at a wavelength of 499 nm and 591.8 nm respectively). In both cases of the higher and lower hybridized modes, the longitudinal electric LSP mode of the dimer is excited (similar to Fig. 2(c)). Note that the two modes differ in phase such that for the higher energy mode, the plasmon oscillation is in phase with the cavity mode (where the dimer is located), while it is antiphase for the lower energy mode. The full width half-maximum (FWHM) for each of the higher and lower energy modes is 19 nm and 15 nm respectively. A 24% increase in enhancement with an enhancement factor of $|{E}_{loc}|/|{E}_{0}|=\,252.6$ is shown at the gap of the dimer for the lower energy hybridized mode. While for the higher energy hybridized mode, the near-field enhancement is $|{E}_{loc}|/|{E}_{0}|=\,228.3$ at the gap of the dimer.

**Figure 5:**
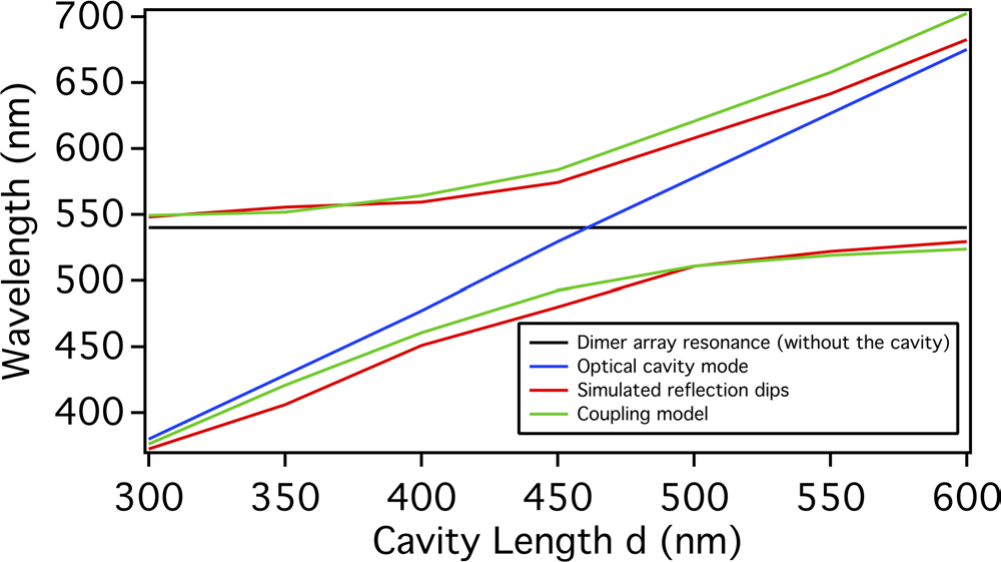
The simulated (red lines) and calculated (green lines) positions of the reflection dips as the cavity’s total length d is varied from 300 nm to 600 nm in steps of 50 nm while all other conditions are kept the same. The calculated reflection dips are found using the coupled oscillator model of the dimers array mode and the optical cavity mode. The coupling strength is 76 meV. The blue line and the horizontal black line represent the cavity modes and the bare (without the cavity) dimers array mode resonance respectively.

In the following we will show that such a high electric field enhancement results from the strong coupling between the collective mode of the dimers array and the optical mode of the FP cavity. This strong coupling results in two hybridized modes, which are the high and low energy hybridized modes whose energies can be calculated using the two-level coupled oscillator model^36, 51, 52^.

$${E}_{+,-}=({E}_{AR}+{E}_{Cavity})/2\pm \sqrt{{\rm{\Delta }}/2+{({E}_{AR}-{E}_{Cavity})}^{2}/4}$$

where ${E}_{AR}$ and ${E}_{Cavity}$ are the energies of the dimers array collective mode and the FP cavity second mode respectively. Δ is the coupling strength. For the FP cavity alone without the dimers array, the resonant condition for the *N*th mode is given by ${\lambda }_{N}=2n(d+\delta )/N$, where *n* is the real part of the refractive index of the material separating the two mirrors, and *d* is the cavity’s total length. $\delta$ is the apparent length increase of the cavity due to the reflection phase at the mirrors. By using FDTD simulations, we determined $\delta =76\,{\rm{nm}}$. ${E}_{AR}$ is also found through numerical simulations as 2.3 eV corresponding to a wavelength of 540 nm as shown in Fig. 2. Figure 5 shows the simulated (red lines) and calculated (green lines) positions of the reflection dips as the cavity’s total length d is varied from 300 nm to 600 nm in steps of 50 nm while all other conditions are kept the same.

The coupling strength in the coupled oscillator model is taken to be 76 meV. Figure 5 shows good agreement between the simulated and calculated reflection dips. The blue line and the horizontal black line represent the cavity modes and the dimers array collective mode resonance respectively. At the crossing of the blue and black lines, the positions of the reflection dips shows a large anticrossing due to the strong coupling between the cavity mode and the array mode. Moving away from the position of the anticrossing, the reflection dips approximately follow one of the two lines.

**Figure 6:**
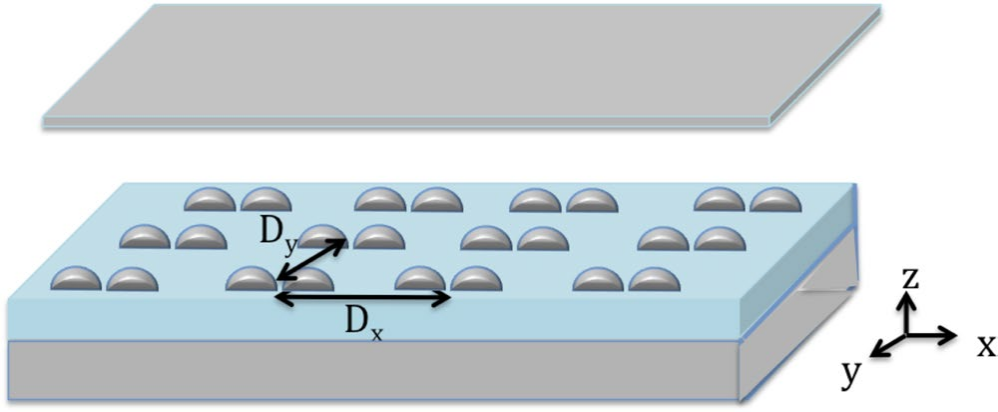
Schematic representation of a hemispherical dimers array on a glass (SiO_2_) substrate of refractive index of 1.45 placed within an FP cavity. Both the dimer and the FP mirrors are silver where the complex refractive index is taken from the data of Palik (0–2 μm)^57^. The surrounding medium is air (index of refraction = 1). The dimers array is in the x-y plane with a periodicity (dimer center-to-center) of D_x_ and D_y_ in the *x* and *y* directions respectively. The FP upper mirror is of a thickness of 15 nm as to allow for the light from the source to pass through it. The lower mirror’s thickness is 500 nm. A TM plane wave source of a wavelength ranging from 520 nm to 850 nm, placed above the FP cavity (above the 15 nm thick mirror), illuminates the array at normal incidence along the *z* direction. The distance between the two mirrors is the total length of the cavity.

To realize a practical implementation of this coupling, the array is placed on a glass (SiO_2_) substrate of index of refraction of 1.45 as in Fig. 6. Below the glass substrate is a 500 nm thick mirror (zero transmission), another mirror of a thickness of 15 nm is placed above the array (the transmissions ranges from 65.3% to 35.5% as the wavelength changes from 520 nm to 850 nm). Both the dimers and the FP mirrors are silver where the complex refractive index is taken from the data of Palik (0–2 μm)^50^ (Note that similar results in terms of the splitting and enhancement can be obtained if Au is used). As in the first model, the surrounding medium is air (index of refraction = 1). The dimers array is in the x-y plane with a periodicity (dimer center-to-center) of D_x_ and D_y_ in the *x* and *y* directions respectively. A TM plane wave source placed above the FP cavity (above the 15 nm thick mirror) illuminates the array at normal incidence along the *z* direction. All other simulation conditions are similar to the first model without using the substrate. Note that because of the glass substrate, the collective resonance of the bare dimers array without the cavity is red-shifted^53–56^ and the diffractive coupling is less efficient due to the inhomogeneous (asymmetric) environment around the particles^24^. However it was shown that for large enough particles, strong diffractive coupling can occur even in an asymmetric configuration^29, 57, 58^. Furthermore, additional peaks at the blue side of the original resonance can arise^59–61^. We first consider the cavity resonances for a FP cavity that is partially filled with glass (SiO_2_) of refractive index of 1.45. Using a similar argument as above, we choose the substrate thickness such that the top of the substrate where the dimers array is placed coincides with the position where the electric field of the FP cavity standing wave is large.

**Figure 7:**
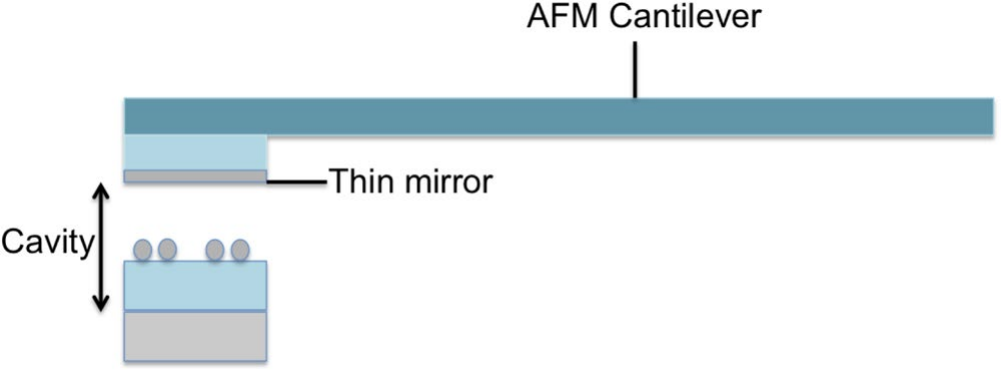
Schematic representation of a possible scheme for implementing the cavity proposed in this paper. The dielectric layer above the thin mirror has a dielectric constant that is close to that of the cantilever (about 1.5).

A possible scheme for implementing the cavity proposed in the paper, is to fabricate the semi-transparent mirror on a AFM cantilever, as in Fig. 7, by means of the two- photon lithography technique^62^. The accurate dynamical distance control that can be reached by AFM in the z direction, the distance between the two cavity mirrors, would be effective for optimizing the resonance at the experimental condition given by the whole device. We believe that this proposed scheme would be successfully compatible with fast scanning techniques where both, high enhancement will be combined with high density distribution of hot spots for light enhanced spectroscopies.

**Figure 8:**
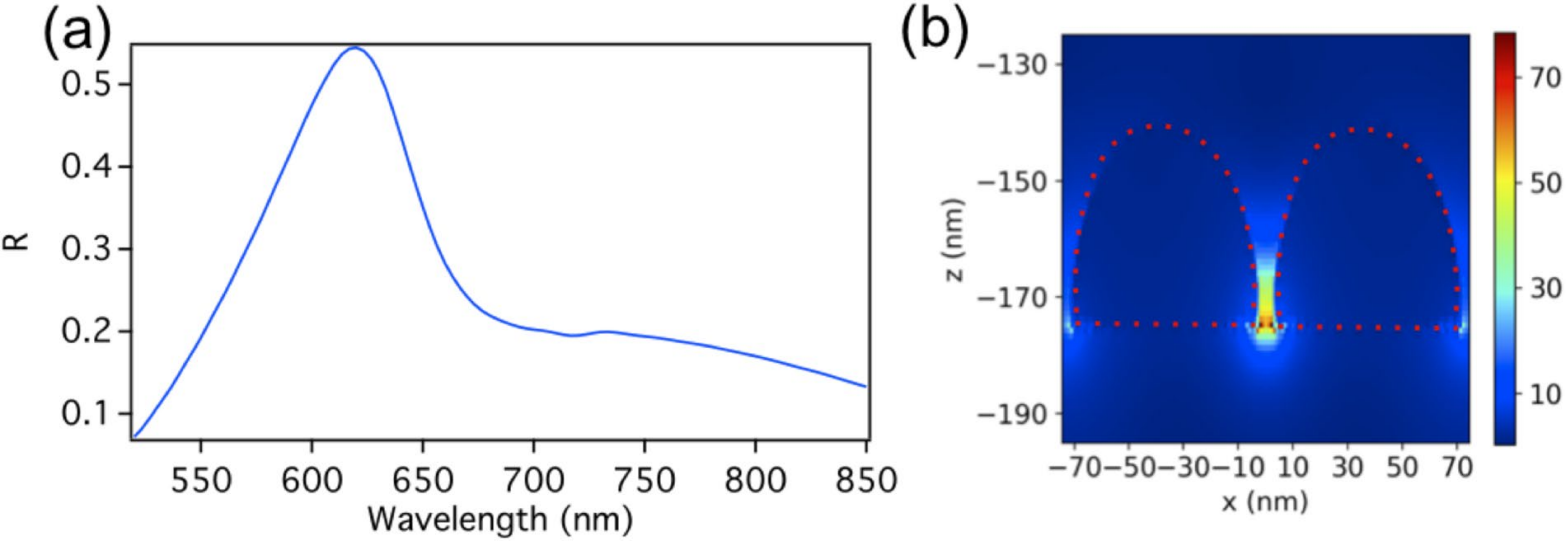
(**a**) The reflection spectrum of the hemispherical dimers array on the glass substrate alone without the FP cavity. A reflection maximum is observed at λ = 620 nm, which is due to the excitation of the dimer’s longitudinal LSP mode enhanced by the far-field diffraction coupling. (**b**) The corresponding near electric field intensity distribution at the reflection peak of λ = 620 nm for a unit cell where an enhancement factor of $|{E}_{loc}|/|{E}_{0}|=78.4$ is observed.

As an example, we consider a hemispherical dimers array where each hemisphere has a radius of 35 nm. The gap between the hemispheres is 5 nm. The glass substrate is of a thickness of 350 nm. The periodicities (dimer center to center) in the *x* and *y* directions are 160 nm and 335 nm respectively. As in the first example of the dimers array without a substrate, the dimer size and the periodicity of the array are chosen such that the collective resonance wavelength of the array matches that of the FP cavity mode (in this case the third order cavity mode). Note that due to the presence of the substrate, the resonance wavelength of a given cavity mode is shifted to a higher value (the second mode is at 903 nm and the third mode is at 1924 nm). A TM plane wave source of a wavelength ranging from 520 nm to 850 nm, placed above the FP cavity (above the 15 nm thick mirror), illuminates the array at normal incidence along the *z* direction. The reflection spectra of the dimer array on the substrate without using the cavity is shown in Fig. 8(a). A reflection maximum is observed at λ = 620 nm, which is due to the excitation of the dimer’s longitudinal electric LSP mode enhanced by the far-field diffraction coupling. We choose the periodicity in *x* and *y* to be 160 nm and 335 nm specifically as to show the splitting more clearly and get a single resonance peak for the dimer array without the cavity. This is because due to the glass substrate, additional peaks at the blue side of the original resonance can arise for arbitrary periods^59–61^. Figure 8(b) shows the corresponding near electric field intensity distribution at the reflection peak for a unit cell of the array on the substrate where an enhancement factor of $|{E}_{loc}|/|{E}_{0}|=78.4$ is observed.

**Figure 9:**
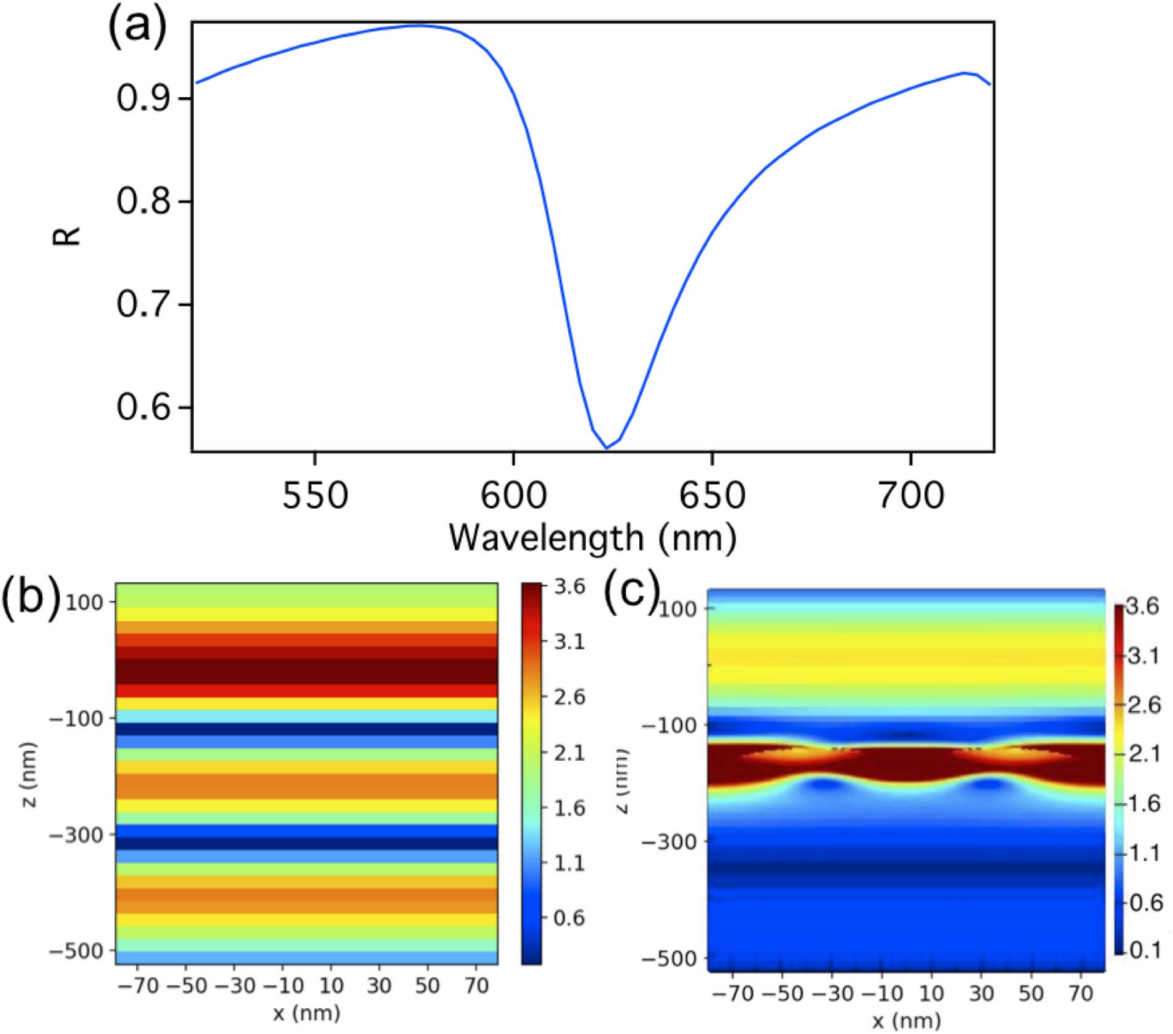
(**a**) The reflection spectra of the FP cavity, containing a substrate, of a total length of d = 657.5 nm without embedding the dimers array. A reflection dip is observed at λ = 623.3 nm resulting from the excitation of the third order cavity mode. (**b**) The corresponding electric field intensity distribution of the FP standing wave of the third order mode (**c**) The electric field intensity of the FP third order standing wave as it interacts with the dimer when the dimer array is placed on the substrate. Note that the field intensity is normalized here to 3.62 V/m as to make the interaction more clear, however the actual field intensity is 102.3 V/m and 112.7 V/m at the dimer gap for the higher and lower energy modes respectively as shown in Fig. 10.

**Figure 10:**
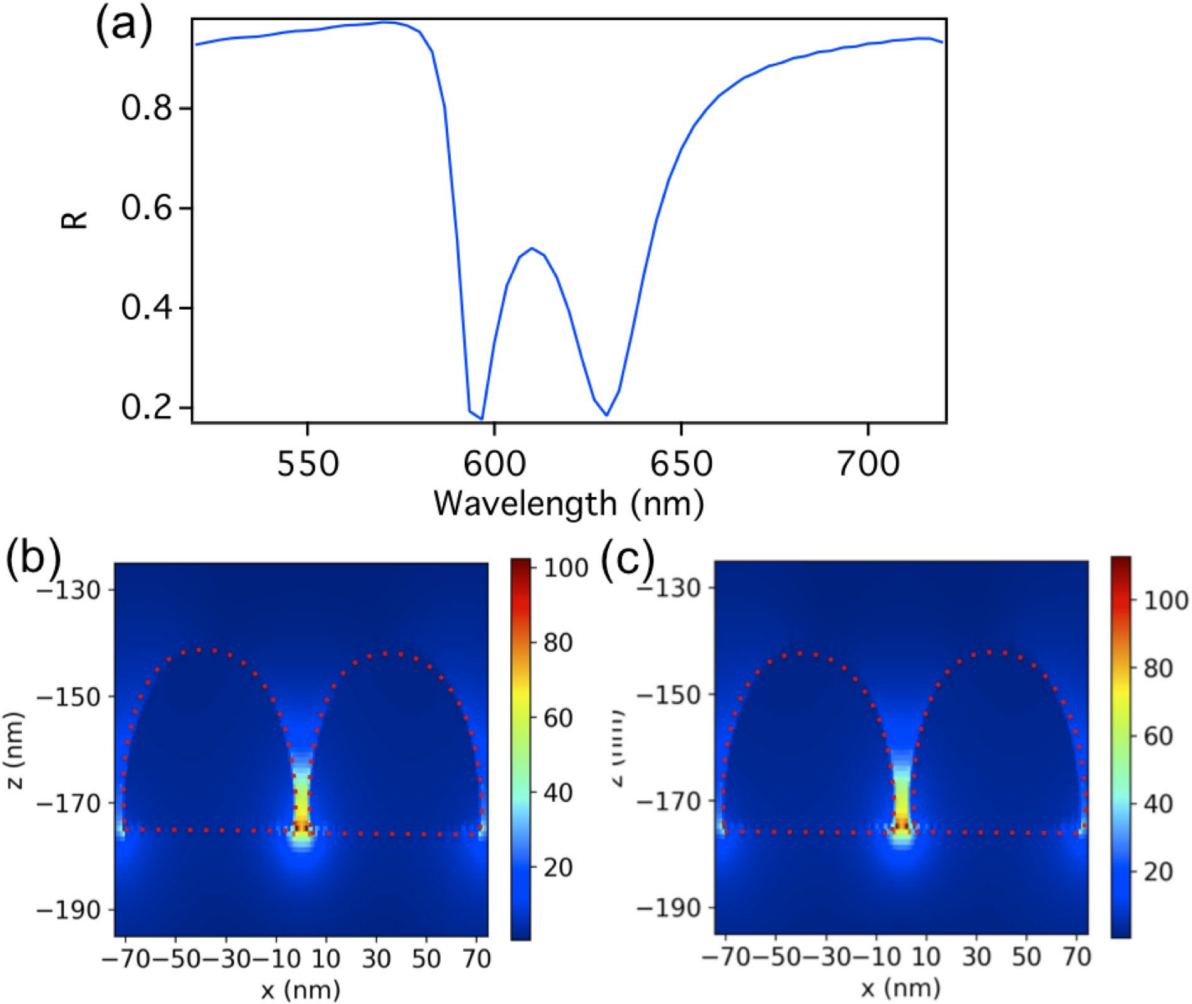
(**a**) Shows the dimers array mode splits into two hybridized modes of narrow bandwidth when the array on the substrate is placed within the cavity of a length of d = 657.5 nm. (**b**) and (**c**) shows the corresponding near electric field intensity enhancement of the unit cell of the dimers array (containing one dimer), when embedded in a FP cavity, for the higher and lower energy modes respectively (at a wavelength of 596.7 nm and 630 nm respectively). An enhancement factor of $|{E}_{loc}|/|{E}_{0}|=102.3$ is shown at the gap of the dimer for the higher energy hybridized mode in (**b**), while for the lower energy hybridized mode in (**c**); the near- field enhancement is $|{E}_{loc}|/|{E}_{0}|=112.7$ at the gap. The full width half-maximum (FWHM) for each of the higher and lower energy dips is 7.5 nm and 12.5 nm respectively.

In Fig. 9(a), we show the reflection spectra of the FP cavity alone where its total length is d = 657.5 nm (thin mirror at 307.5 nm above the substrate) that is partially filled with a glass substrate (index of refraction of 1.45) of thickness of 350 but without the dimers array (as in Fig. 6 excluding the dimers). A reflection dip is observed at λ = 623.3 nm resulting from the excitation of the third order cavity mode. Figure 9(b) shows the electric field intensity distribution for the FP standing wave of the third order mode at 623.3 nm. The dimer array is then placed within this cavity. The top of the substrate where the dimers array is placed coincides with the position where the electric field of the FP cavity standing wave is large. Figure 9(c) shows the electric field intensity of the FP third order standing wave as it interacts with the dimer (when the dimer array is placed within the cavity on top of the substrate). Note that the electric field intensity is normalized here to 3.62 V/m as to make the interaction between the dimer and the cavity more clear, however the actual field intensity is 102.3 V/m and 112.7 V/m at the dimer gap for the higher and lower energy modes respectively as shown in Fig. 10.

In Fig. 10(a), it is shown the dimers array mode splits into two hybridized modes of narrow bandwidth when the array is placed within the cavity. Figure 10(b) and (c) shows the corresponding near electric field intensity enhancement of the unit cell (containing one dimer) of the dimers array on the substrate, when embedded in the FP cavity, for the higher and lower energy modes respectively (at the wavelengths of 596.7 nm and 630 nm respectively). As is the case for the dimer array without the substrate, the longitudinal electric LSP mode of the dimer is excited for both the higher and lower energy modes. Note again, as in the first case of the array without the substrate, that the two modes differ in phase such that for the higher energy mode, the plasmon oscillation is in phase with the cavity mode, while it is antiphase for for the lower energy mode. The full width half-maximum (FWHM) for each of the higher and lower energy dips is 7.5 nm and 12.5 nm respectively. An enhancement factor of $|{E}_{loc}|/|{E}_{0}|=102.3$ is shown at the gap of the dimer for the higher energy hybridized mode. While for the lower energy hybridized mode, the near- field enhancement is $|{E}_{loc}|/|{E}_{0}|=112.7$ at the gap, which is a 43% increase in enhancement relative to the enhancement without using the cavity.

The wavelength in Table 1. refers to the wavelength at maximum enhancement when the cavity is introduced. For the Al dimer sphere, each sphere has a radius of 35 nm, while for the Ag dimer spheres; each sphere has a radius of 40 nm. For the Ag bow-tie dimer, each is an equatorial triangle with a side of 70 nm and a thickness of 20 nm. Finally for the Au rounded rods dimer, the radii are 30 nm and 30 nm, and the total length of the rod is 100 nm. Note that we chose the sizes of the different dimers, as well as the periodicity in both *x* and *y*, such that the collective resonance wavelength of the dimers array matches that of the FP cavity resonance.

**Table 1:** Shows different arrangements with different dimer shapes, different materials and other parameters including the total length of the cavity, the thickness of the substrate, the periodicity in *x* and *y*, the enhancement at the gap of the dimer with and without the FP cavity, the gap size and the mode order of the cavity to produce high enhancement at the wavelengths of 492.2 nm, 521.2 nm, 677 nm, 686.4 and 750 nm, where an increase in enhancement of 81%, 25%, 44%, 54% and 200% is observed respectively.

Wavelength (nm)	Dimer Material	Dimer Shape	D_x_ (nm)	D_y_ (nm)	Cavity Mode order	Substrate Thickess (nm)	Cavity length d (nm)	$|{{\boldsymbol{E}}}_{{\boldsymbol{loc}}}|/|{{\boldsymbol{E}}}_{{\rm{0}}}|$ without the cavity	$|{{\boldsymbol{E}}}_{{\boldsymbol{loc}}}|{\boldsymbol{/}}|{{\boldsymbol{E}}}_{{\bf{0}}}|$ with the cavity	Gap size (nm)
492.9	Al	spheres	160	335	4	350	679.5	37.8	68	5
521.2	Ag	spheres	240	370	4	350	717.5	72	90	5
677	Ag	Bow-tie	365	395	3	350	792.5	200	288	5
686.4	Au	Rounded rods	270	370	3	350	757.5	58.5	90	5
750	Au	Bow-tie	365	395	3	350	867.5	128.25	255.6	5

The details of the dimensions of each dimer are given below the table. Note that the Ag Bow-tie configuration produces a two-fold enhancement.

## Conclusion

We theoretically present an approach to enhance the near-field intensity at a plasmonic dimer gap (hot spot) through coupling the electric localized surface plasmon (LSP) resonance of a dimer with the resonant modes of a Fabry-Perot (FP) cavity. The strong coupling is demonstrated by the anticrossing in the reflection spectra and a Rabi splitting of 76 meV. Up to 2-fold increase in enhancement can be achieved compared to the enhancement without using the cavity. In addition, the resonance splitting allows for greater flexibility in using the same array at different wavelengths. We then further propose a possible scheme for implementing the cavity. In addition, we show different configurations using different dimer shapes, materials and cavity lengths to maximize the near-field enhancement at the wavelengths of 492.9 nm, 521.2 nm, 686.8 nm, 677 nm and 750 nm. Such high field enhancement has potential applications in many optical devices, such as in sensors and high resolution imaging devices. Finally this array-cavity system is simple with fixed and reproducible geometries that give rise to a high and stable Raman enhancement, which makes it a good candidate for the practical realization of SERS-based sensors.

## Methods

### FDTD simulations

Simulations for the reflection spectra as well as the near field enhancement were carried out using the commercial software (Lumerical Solutions Inc. Ver. 8.7.0). Perfectly matched layer boundary conditions (PML) where used above and below the array in the z-direction to absorb scattered waves at the boundaries of the simulation domain. The mesh accuracy is set to 3, corresponding to a λ/14 step size. A mesh override region of 1 nm in all three directions is used around the dimer for a better resolution of the gap’s near-field enhancement. To simulate an infinite array of the unit cell, periodic boundary conditions are used in both *x* and *y* directions.
